# Targeting mTOR signaling pathways in multiple myeloma: biology and implication for therapy

**DOI:** 10.1186/s12964-024-01699-3

**Published:** 2024-06-11

**Authors:** Yanmeng Wang, Niels Vandewalle, Kim De Veirman, Karin Vanderkerken, Eline Menu, Elke De Bruyne

**Affiliations:** 1 Translational Oncology Research Center (TORC) – Team Hematology and Immunology (HEIM), Vrije Universiteit Brussel (VUB), Jette, Belgium; 2 Translational Oncology Research Center (TORC) – Team Hematology and Immunology (HEIM), Universitair Ziekenhuis Brussel (UZ Brussel), Jette, Belgium

**Keywords:** mTOR, Multiple myeloma, Protein synthesis, Targeted therapy

## Abstract

Multiple Myeloma (MM), a cancer of terminally differentiated plasma cells, is the second most prevalent hematological malignancy and is incurable due to the inevitable development of drug resistance. Intense protein synthesis is a distinctive trait of MM cells, supporting the massive production of clonal immunoglobulins or free light chains. The mammalian target of rapamycin (mTOR) kinase is appreciated as a master regulator of vital cellular processes, including regulation of metabolism and protein synthesis, and can be found in two multiprotein complexes, mTORC1 and mTORC2. Dysregulation of these complexes is implicated in several types of cancer, including MM. Since mTOR has been shown to be aberrantly activated in a large portion of MM patients and to play a role in stimulating MM cell survival and resistance to several existing therapies, understanding the regulation and functions of the mTOR complexes is vital for the development of more effective therapeutic strategies. This review provides a general overview of the mTOR pathway, discussing key discoveries and recent insights related to the structure and regulation of mTOR complexes. Additionally, we highlight findings on the mechanisms by which mTOR is involved in protein synthesis and delve into mTOR-mediated processes occurring in MM. Finally, we summarize the progress and current challenges of drugs targeting mTOR complexes in MM.

## Introduction

Multiple Myeloma (MM) is a hematological malignancy characterized by the accumulation of abnormal monoclonal plasma cells in the bone marrow (BM). It is the second most frequent hematological cancer and comprises 10% of all hematological malignancies, with defined clinical characteristics including hypercalcemia, renal failure, anemia, and bone lesions (CRAB) [1, 2]. Worldwide, an estimated 160,000 people were diagnosed with MM in 2020 [3].

The discovery of novel drugs, including proteasome inhibitors (PI; Bortezomib, Carfilzomib, and Ixazomib) and immunomodulatory drugs (IMiD; Thalidomide, Lenalidomide and Pomalidomide), has significantly altered the therapeutic landscape for MM in both the frontline and relapsed/refractory setting during the past two decades. The combined application of these drugs, together with the use of myeloablative chemotherapy and autologous stem cell transplantation (ASCT), has translated into prolonged overall survival (OS) rates with reduced toxicity and improved quality of life [4, 5]. More recently, immunotherapy has emerged as a powerful new tool to obtain durable responses in MM. This type of therapy includes monoclonal antibodies, immune checkpoint inhibitors, bispecific antibodies, chimeric antigen receptor T (CAR-T) cells, and peptide vaccines [6–9]. However, despite these new advancements, MM remains largely incurable due to either the occurrence of immune suppression or the development of drug resistance to multiple drug classes. With modern therapy, the first relapse typically occurs after about 3–4 years following initial diagnosis [2].

The (hypoxic) BM environment wherein the MM cells grow provides support and protection against different types of drugs. It consists of several cell types including BM stromal cells, endothelial cells, osteoclasts and osteoblasts. All these different cell types contribute to the growth and expansion of the MM clone, by providing nutrients and growth factors such as metabolites, amino acids, and cytokines. The main growth factors for MM cells include interleukin-6 (IL-6), insulin-like growth factor-1 (IGF-1) and vascular endothelial growth factor (VEGF). These growth factors will activate different signaling cascades with the ultimate goal to stimulate biogenesis and cell division [10].

Maintaining a stable proteome is essential for the growth and survival of every cell, yet protein synthesis (mRNA translation) and folding processes are inherently error-prone. The key steps in protein synthesis include initiation, elongation, termination and ribosome recycling [11]. Excessive protein synthesis has been associated with human cancers with elevated global translation, such as MM where there is a high production of immunoglobulins. The mammalian target of rapamycin (mTOR) kinase controls several factors involved in protein synthesis and aberrant mTOR activation through various mechanisms is frequently observed in a large portion of MM patients, contributing to cell survival, growth and drug resistance [12–15]. Moreover, accumulating research provides evidence that targeting the mTOR pathway can restrict protein synthesis in MM, resulting in cell death. Therefore, protein synthesis in general and the mTOR pathway specifically both represent interesting (new) targets in MM. This review will provide an update on what is known about the dysregulation of the mTOR pathway in MM and discuss promising new therapeutic strategies.

### Overview of the mTOR pathway

#### Structure of the mTOR complexes

**Fig. 1:**
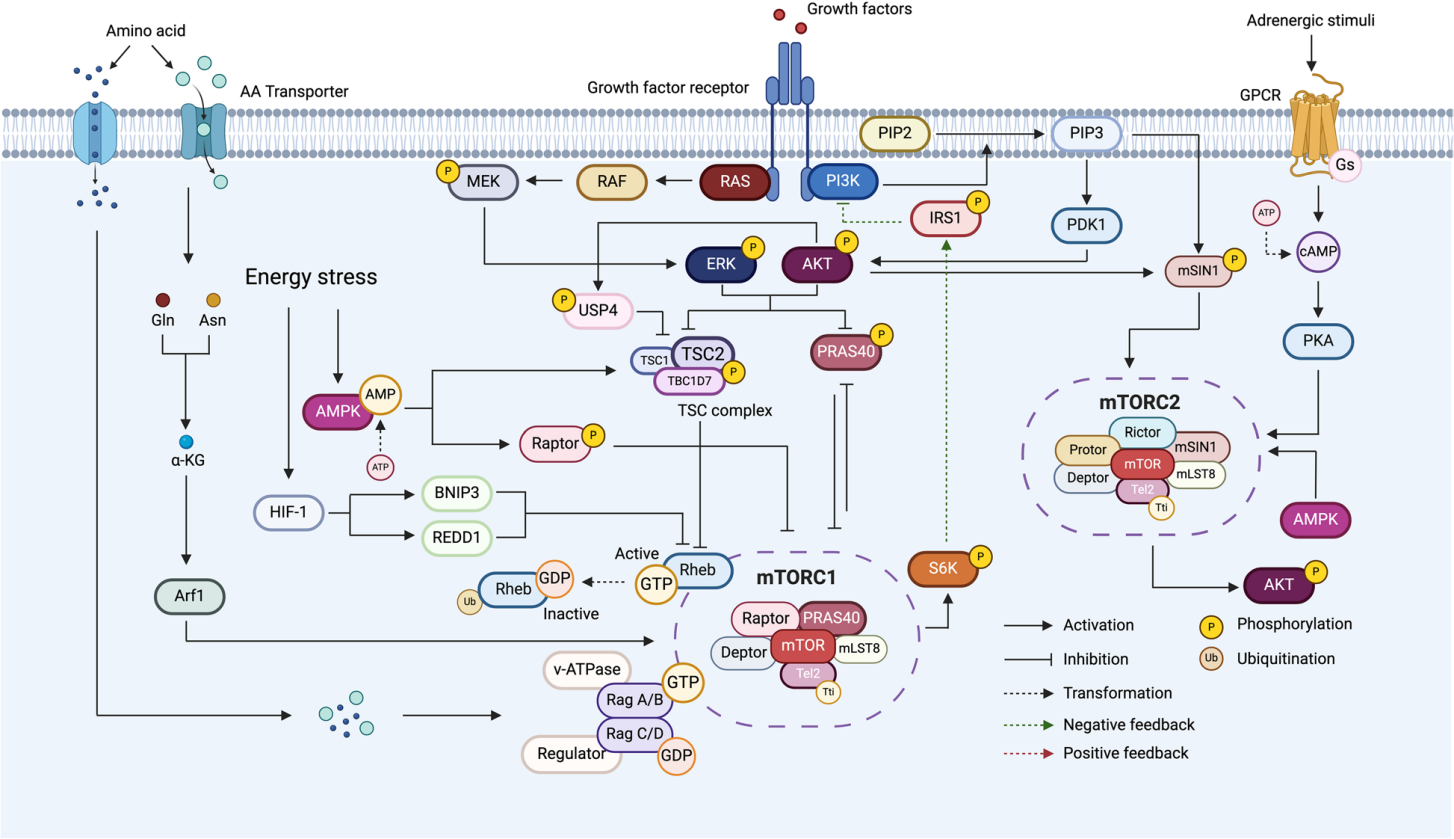
Schematic representation of the mTOR signaling pathway. mTORC1 and mTORC2 share mTOR, Deptor, mLST8, Tel2 and Tti1, while Raptor and PRAS40 are unique for mTORC1 and Rictor, mSIN1, and Protor are unique for mTORC2. Growth factors stimulate PI3K to convert PIP2 to PIP3. PIP3 will then recruit PDK1, leading to phosphorylation of AKT. In addition, RAS signaling can also be activated by growth factors, promoting the activation of RAF/MEK/ERK pathway. Activated AKT and/or ERK will then phosphorylate the TSC complex and/or PRAS40, leading to the relief of their mTORC1 inhibitory activity. For the TSC complex, phosphorylation by AKT will inhibit its GAP activity towards Rheb, allowing GTP-bound Rheb to bind to and activate mTORC1. Amino acids stimulate mTORC1 by promoting the formation of Rags-v-ATPase-Regulator complexes. In addition, Gln and Asn activate mTORC1 in a RAG-independent manner via the small GTPase Arf1. In contrast, energy stress will suppress mTORC1 activity by activating AMPK, resulting in the subsequent inhibition of Raptor and activation of the TSC complex. In addition, HIF-1 will prevent mTORC1 activation by inducing BNIP3 and/or REDD1, leading to Rheb inactivation. As for mTORC2, growth factors directly phosphorylate mSIN1 in a PIP3-dependent manner or through partially activated AKT, thereby promoting mTORC2 activation. Gs-coupled β2-adrenoceptor also promotes mTORC2 activation, by stimulating cAMP accumulation and PKA activation. In addition, AMPK directly activates mTORC2. In contrast, mTORC1 inhibits mTORC2 activation, by negatively regulating PI3K/AKT signaling through S6K1. mTORC1, mTOR complex 1; mTOR, Mammalian target of rapamycin; Raptor, Regulatory-associated protein of mTOR; Deptor, DEP-domain containing mTOR-interacting protein; PRAS40, Proline-rich AKT substrate 40 kDa; mLST8, Mammalian Lethal with Sec-13 protein 8; Rictor, Rapamycin-insensitive companion of mTOR; mSIN1, Mammalian stress-activated map kinase-interacting protein 1; Protor, Protein observed with rictor; Tel2, Telomere maintenance 2; Tti1, Tel2 interacting protein 1; PI3K, Phosphatidylinositol-3-kinase; PIP2, phosphatidylinositol-4,5-bisphosphate; PIP3, Phosphatidylinositol-3, 4, 5-triphosphate; PDK1, Phosphoinositide-dependent kinase 1; AKT, Protein kinase B; MEK, Mitogen-activated protein kinase; ERK, Extracellular-signal-regulated kinase; TSC, Tuberous sclerosis; GAP, GTPase-activating protein; Gln, Glutamine; Asn, Asparagine; Arf1, ADP-ribosylation factor 1; AMPK, Adenosine 5'-monophosphate-activated protein kinase; HIF-1, Hypoxia inducible factor 1; BNIP3, BCL2-interacting protein 3; REDD1, DNA damage inducible transcript 4; S6K1, Ribosomal S6 kinase; PKA, cAMP-dependent protein kinase

TOR is an evolutionarily conserved Ser/Thr-protein kinase that exists in two structurally and functionally distinct complexes, namely mTOR complex 1 (mTORC1), sensitive to the macrolide fungicide rapamycin, and the insensitive mTORC2 complex. They are both large complexes composed of multiple proteins. A regulatory-associated protein of mTOR (Raptor) and proline-rich AKT substrate 40 kDa (PRAS40) are specific to mTORC1, whereas mammalian stress-activated map kinase-interacting protein 1 (mSIN1), rapamycin-insensitive companion of mTOR (Rictor) and protein observed with rictor (Protor) 1 and 2 are exclusive components of mTORC2 (Fig. 1). However, they share mTOR, mammalian Lethal with Sec-13 protein 8 (mLST8), DEP-domain containing mTOR-interacting protein (Deptor) and the Telomere maintenance 2 (Tel2) and Tel2 interacting protein 1 (Tti1) complex.

As a subunit of mTORC1, Raptor plays a crucial role in controlling the stability, lysosome surface localization, substrate recognition and function of mTORC1 [16–20]. By contrast, PRAS40 is recognized as an intrinsic inhibitory component of mTORC1, which binds to Raptor and competes with other substrates for mTORC1 binding, thereby inhibiting downstream signaling [21–23].

While mTORC1 has been well characterized in the last decade, knowledge on mTORC2 is only now rapidly developing. As a central member of the mTORC2 complex, mSIN1 contains an N-terminal domain (NTD), a RAS-binding domain (RBD), a conserved region in the middle (CRIM), and a pleckstrin homology (PH) domain in its C-terminal region. Both the RBD domain, through its interaction with active RAS, and the PH domain account for mTORC2 activation [24, 25], while the CRIM domain is in charge of mTORC2 substrate recruitment [26–28]. In addition, mSIN1 directly interacts with Rictor through its NTD, connecting Rictor with mLST8 to stabilize the mTORC2 complex [28, 29]. Rictor has comparable functions as Raptor, controlling mTORC2’s assembly, stability, and activity [30], whereby its C-terminal domain is responsible for mTORC2’s insensitivity to rapamycin [28]. Protor consists of two isoforms which also interact with Rictor through a conserved N-terminal region [31, 32], however, their role remains unclear.

When evaluating the shared components, mLST8 appears to be more important for the mTORC2 complex than the mTORC1 complex. Knockdown of mLST8 blocks activation of the mTORC2 substrates, while retaining the ability to phosphorylate mTORC1 substrates [33]. Studies indicate that this is mediated by interacting with the mTORC2 cofactors Rictor and mSIN1, thereby enhancing the assembly of the complex [34]. The stabilizing proteins Tel2 and Tti1 constitutively interact with mTOR in both mTORC1 and mTORC2, and the knockdown of either Tti1 or Tel2 results in the disassembly of both complexes [35]. Finally, Deptor is a highly conserved protein that binds to mTOR through its PDZ domain, thereby inhibiting the activity of both mTORC1 and mTORC2. However, Deptor and mTOR can also regulate each other, whereby mTOR kinase activity will phosphorylate Deptor, thereby promoting its release from mTOR and reversing its activity [36].

#### Regulation of the mTOR complexes

The activity of mTORC1 is regulated by several factors, including growth factors, amino acids, stress signals and cellular energy (Fig. 1). Several growth factors can activate mTORC1 by interacting with their cell-surface receptor tyrosine kinase(s), leading to the activation of the phosphatidylinositol-3-kinase (PI3K)/AKT and RAS/ERK (extracellular-signal-regulated kinase) pathways [37, 38]. By blocking either the tuberous sclerosis (TSC) complex or PRAS40, two mTORC1 negative regulators, AKT and ERK both positively control mTORC1 activity [39–41]. The TSC complex, which consists of three core subunits, TSC1, TSC2, and TBC1D7, keeps the small G-protein Rheb in an inactive state via its GTPase-activating protein (GAP) activity and by promoting Rheb ubiquitination [42, 43]. However, upon growth factor stimulation, AKT will phosphorylate both TSC2 and the deubiquitinase ubiquitin specific peptidase 4 (USP4), resulting in the release of Rheb from the inhibitory effect of the TSC complex [44]. PRAS40 is not only a component of mTORC1, but also a substrate of mTORC1, located downstream of mTORC1 but upstream of its effectors. Therefore, it can be controlled by both AKT or mTORC1 itself. While activated AKT dissociates PRAS40 from the mTORC1 complex by phosphorylating its threonine residue (Thr246), mTORC1 directly phosphorylates PRAS40 at serine residues (Ser183 and Ser221) to impair its inhibitory action [45–47].

It is generally believed that amino acid signaling stimulates mTORC1 activity by regulating its subcellular localization, and Rag guanosine triphosphatases (Rags or Rag GTPases) play a crucial role in this process [48, 49]. When amino acids are sufficiently present, active Rags form a complex with v-ATPase-Regulator and transmit amino acid signaling to the mTORC1 pathway by binding to Raptor. This process recruits mTORC1 to the lysosomal membranes, where Rheb is present, and stimulates mTORC1 activation [50, 51]. While most amino acids activate mTORC1 through Rags, glutamine (Glu) and asparagine (Asn) appear to activate mTORC1 in a Rag-independent manner that requires the small GTPase ADP-ribosylation factor 1 (Arf1) [52]. However, the glutamine sensor and other components involved in this Rag-independent pathway in mammals remain to be studied.

Energy stress controls mTORC1 activation primarily through an adenosine 5'-monophosphate-activated protein kinase (AMPK)-dependent mechanism. Under energy stress, such as glucose deprivation, the concentration of ATP drops dramatically while the cellular levels of AMP and ADP increase. AMP binds to the γ-subunit of AMPK contributing to its activation. AMPK then transmits the energy stress signal to mTORC1 mainly through two mechanisms [41, 53]. Firstly, AMPK activates the TSC complex, which in turn represses Rheb, thereby reducing mTORC1 activity [54, 55]. Secondly, AMPK will directly phosphorylate mTOR and Raptor, which also appears to be required for energy stress-induced inhibition of mTORC1 [56–58]. Additionally, AMPK-independent mechanisms have also been discovered to regulate mTORC1 activity upon stress. For example, mTORC1 can also be inactivated by hypoxia inducible factor 1 (HIF-1), the master regulator of the cellular response to hypoxia. HIF-1, either by inducing BCL2-interacting protein 3 (BNIP3) or by activating DNA damage inducible transcript 4 (DDIT4/REDD1), prevents activation of mTORC1 via direct interaction with Rheb [59–62].

In comparison to mTORC1, the signals activating mTORC2 and the mechanisms involved are less understood and more complicated. Similar to mTORC1, it is generally believed that growth factor-dependent mTORC2 activation requires PI3K/PIP3. In the unstimulated state, the mSIN1 PH domain is bound to the catalytic core within mTOR, thereby impairing mTORC2 activity. Following growth factor stimulation, PIP3 not only recruits Phosphoinositide-dependent kinase 1 (PDK1) and AKT from the cytosol, it will also bind to mSIN1 to expose the catalytic core within mTOR. AKT, which is partially activated through phosphorylation of Thr308 by PDK1, will then phosphorylate mSIN1 at Thr86, leading to a conformational change and subsequent promotion of mTORC2 activity. mTORC2 will then on its turn phosphorylate AKT at Ser473, resulting in full AKT activation [63, 64]. Additional stimuli that can trigger mTORC2 activation include adrenergic signaling via G-protein coupled receptors (GPCR), such as the β2‐adrenoceptor, which stimulates cAMP accumulation and activation of cAMP-dependent protein kinase (PKA), leading to phosphorylation of mTORC2 [65]. Also, AMPK appears to be sufficient to increase mTORC2 catalytic activity towards AKT in an mTORC1-independent manner [66]. Finally, mTORC2 activity is negatively regulated by mTORC1. Elevated mTORC1 activity upon insulin/ IGF-1signaling increases the activity of one of its direct effectors, S6K1 (see below), which in turn will phosphorylate insulin receptor substrate 1 (IRS1) on various negative regulatory sites, thereby inhibiting PI3K signaling and dampening mTORC2 [67].

### Molecular mechanisms of mTOR-mediated translational control

**Fig. 2:**
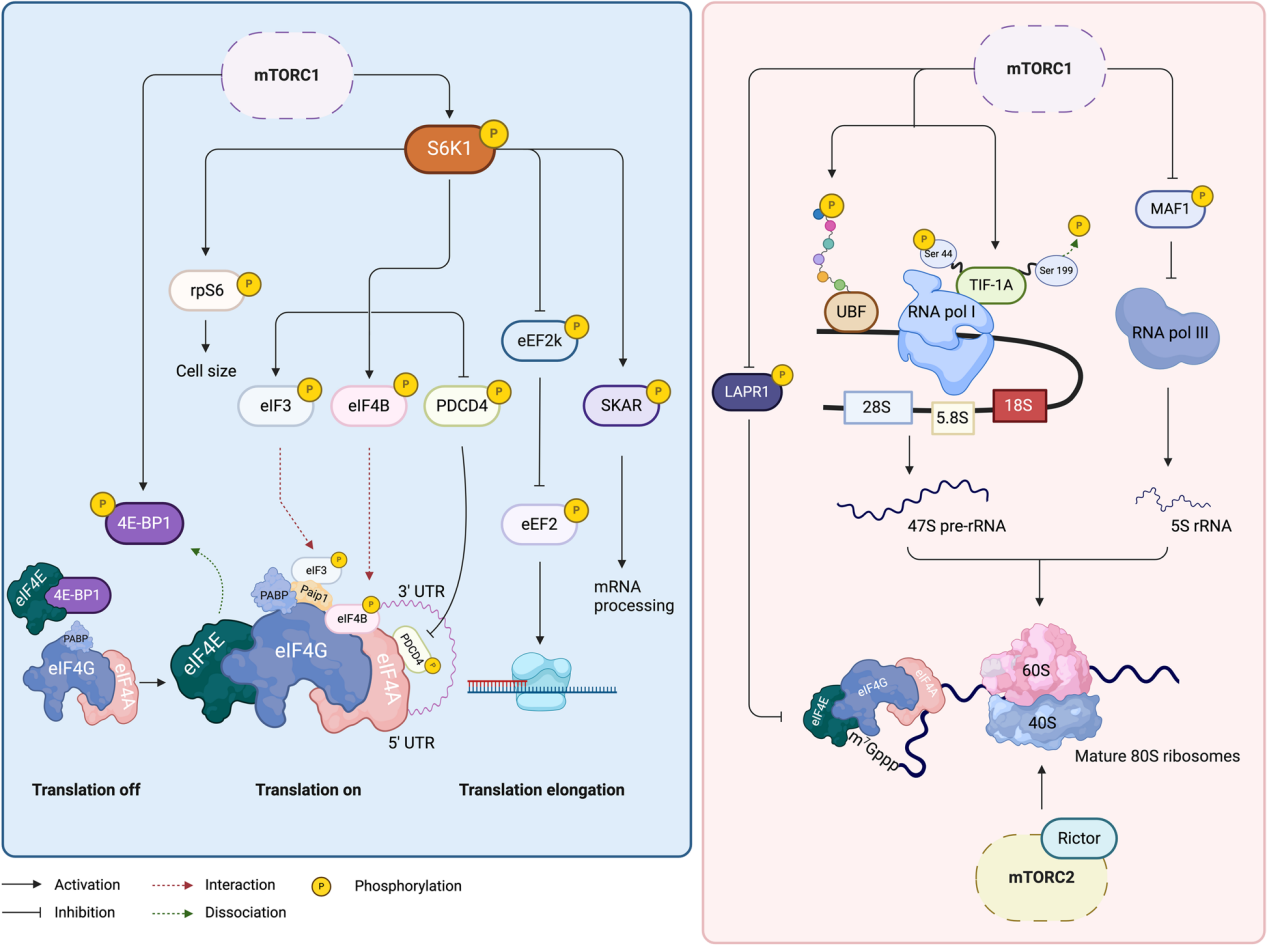
mTOR signaling and regulation of mRNA translation. mTOR signaling controls protein synthesis via regulation of mRNA translation initiation and ribosome biogenesis. mTORC1 phosphorylates 4E-BP1, resulting in the assembly of the eIF4F translation initiation complex. In addition, mTORC1 will phosphorylate S6K1, thereby promoting translation via phosphorylation of rpS6, eIF4B, PDCD4, eIF3, SKAR, and eEF2. In addition, mTORC1 also regulates ribosome biogenesis by activating UBF and TIF-1A, while inhibiting MAF1, thereby modulating Pol I and Pol III transcription. In addition, mTORC1 promotes translation of 5'-TOP transcripts by phosphorylating LARP1. Finally, mTORC2 also regulates ribosome biogenesis by relocating Rictor to the ER. mTOR, Mammalian target of rapamycin; 4E-BP1, Eukaryotic translation initiation factor 4E-binding protein 1; eIF4F, Eukaryotic translation initiation factor 4F; S6K1, Ribosomal S6 kinase 1; mTORC1, mTOR complex 1; rpS6, Ribosomal protein S6; eIF4B, Eukaryotic translation initiation factor 4B; PDCD4, Programmed cell death protein 4; eIF3, Eukaryotic translation initiation factor 3; SKAR, S6K1 Aly/REF-like substrate; eEF2, Eukaryotic elongation factor 2; Pol I/III, RNA polymerase I/III; LARP1, La-related protein 1; mTORC2, mTOR complex 1; Rictor, Rapamycin-insensitive companion of mTOR; 5’-TOP, 5’-terminal oligopyrimidine; ER, Endoplasmic reticulum

mTOR functions as a central coordinator of cellular metabolic homeostasis in response to nutrient levels and growth signals. When ample nutrients and growth factors are present, the activation of the mTOR pathway promotes anabolic pathways, including protein and lipid synthesis, while also stimulating glycolysis and mitochondrial metabolism. Conversely, under conditions of hypoxia or energetic stress, mTOR signaling is inhibited, halting energy-consuming anabolic pathways and promoting catabolic pathways, such as autophagy [68]. In this review, we will discuss how mTORC1 and mTORC2 are involved in multiple aspects of protein synthesis, including activation of the substrates involved in mRNA translation initiation and promotion of ribosome biogenesis (Fig. 2).

#### Activation of mRNA translation initiation

When sufficient nutrients are present, mTORC1 is strongly activated, promoting protein synthesis by phosphorylating eukaryotic initiation factor 4E-binding protein 1 (4E-BP1) and p70 S6 kinase 1 (p70-S6K, also known as S6K1) in a Raptor-dependent manner [69].

To initiate mRNA translation, the mRNA first needs to be unwound or activated by the eIF4F complex, comprising the cap-binding protein eukaryotic translation initiation factor 4E (eIF4E), the RNA helicase eIF4A, and the scaffold protein eIF4G, together with the assistance of eIF4B, eIF3 and poly(A)-binding protein (PABP). In its unphosphorylated state, 4E-BP1 represses translation by binding to and sequestering eIF4E, thereby preventing its interaction with eIF4G. mTORC1 phosphorylates 4E-BP1 at several sites, causing the dissociation of 4E-BP1 from eIF4E [70]. The release of eIF4E enables association with eIF4G and the assembly of the eIF4F translation initiation complex at the 5′end of the mRNA [71]. S6K1 is the second well-established downstream effector of mTOR that is directly phosphorylated by mTOR [72, 73]. S6K1 phosphorylates several factors participating in protein synthesis, including eIF4B, programmed cell death protein 4 (PDCD4), eIF3, eEF2, 40S ribosomal protein S6 (rpS6), and S6K1 Aly/REF-like target (SKAR). Phosphorylation of eIF4B leads to its binding with eIF4G and eIF4A, while phosphorylation of PDCD4 leads to its release from eIF4A, allowing eIF4A to interact with eIF4G. Importantly, eIF4B and PDCD4 phosphorylation by S6K1 is sufficient to maintain protein synthesis, even in the absence of 4E-BP1 [74]. Phosphorylated eIF3 will bind to the PABP regulatory protein PABP-interacting protein 1 (Paip1), thereby stabilizing the interaction between PABP and eIF4G, thus further stimulating translation [75, 76]. The protein kinase eukaryotic elongation factor 2 kinase (eEF2k) is a negative regulator of eEF2, which becomes inhibited after phosphorylation by S6K1, thereby releasing eEF2 and allowing proper elongation [77]. Phosphorylation of rpS6 has been shown to control cell size, however its function in protein synthesis remains elusive [78]. Finally, by interacting with SKAR, S6K1 is recruited to newly synthesized mRNAs in a splicing-dependent manner [79].

#### Ribosome biogenesis

To cope with increased protein synthesis, mTORC1 also promotes several steps in ribosome biogenesis, including ribosomal RNA transcription, synthesis of ribosome proteins and other components required for ribosome assembly. In mammals, the ribosomes contain 4 different rRNAs involved in ribosome assembly, which are transcribed by either RNA polymerase I (Pol I) or RNA polymerase III (Pol III) [80]. Several basal factors required for Pol I-mediated transcription are regulated by mTORC1. Firstly, mTORC1 activates Pol I-mediated transcription by increasing the expression and phosphorylation of UBF, thereby facilitating the recruitment of Pol I to rDNA [81]. Secondly, mTORC1 activates TIF-1A, a transcription factor that connects Pol I with UBF to initiate the transcription of pre-ribosomal RNA [82]. Thirdly, MAF1 is a key repressor of Pol III transcription, which becomes inhibited after phosphorylation by mTORC1 [83]. In addition, mTORC1 also controls the translation of a variety of mRNAs, particularly the 5’-terminal oligopyrimidine (5’-TOP) transcripts encoding ribosomal proteins, via direct phosphorylation of the La-related protein 1 (LARP1), a repressor of ribosomal protein mRNA translation [84]. Phosphorylation of LARP1 abolishes its blockage on the assembly of the eIF4F complex [85, 86]. Of note, enhanced ribosome biogenesis facilitates the transition of cells from an epithelial to a mesenchymal state, a process known as epithelial-mesenchymal transition (EMT). This EMT-associated ribosome biogenesis is accompanied by a pronounced increase in Rictor’s localization in the endoplasmic reticulum (ER), indicating also a regulatory role of mTORC2 in ribosome biogenesis [87].

### Aberrant mTOR pathway signaling in MM cells

**Fig. 3:**
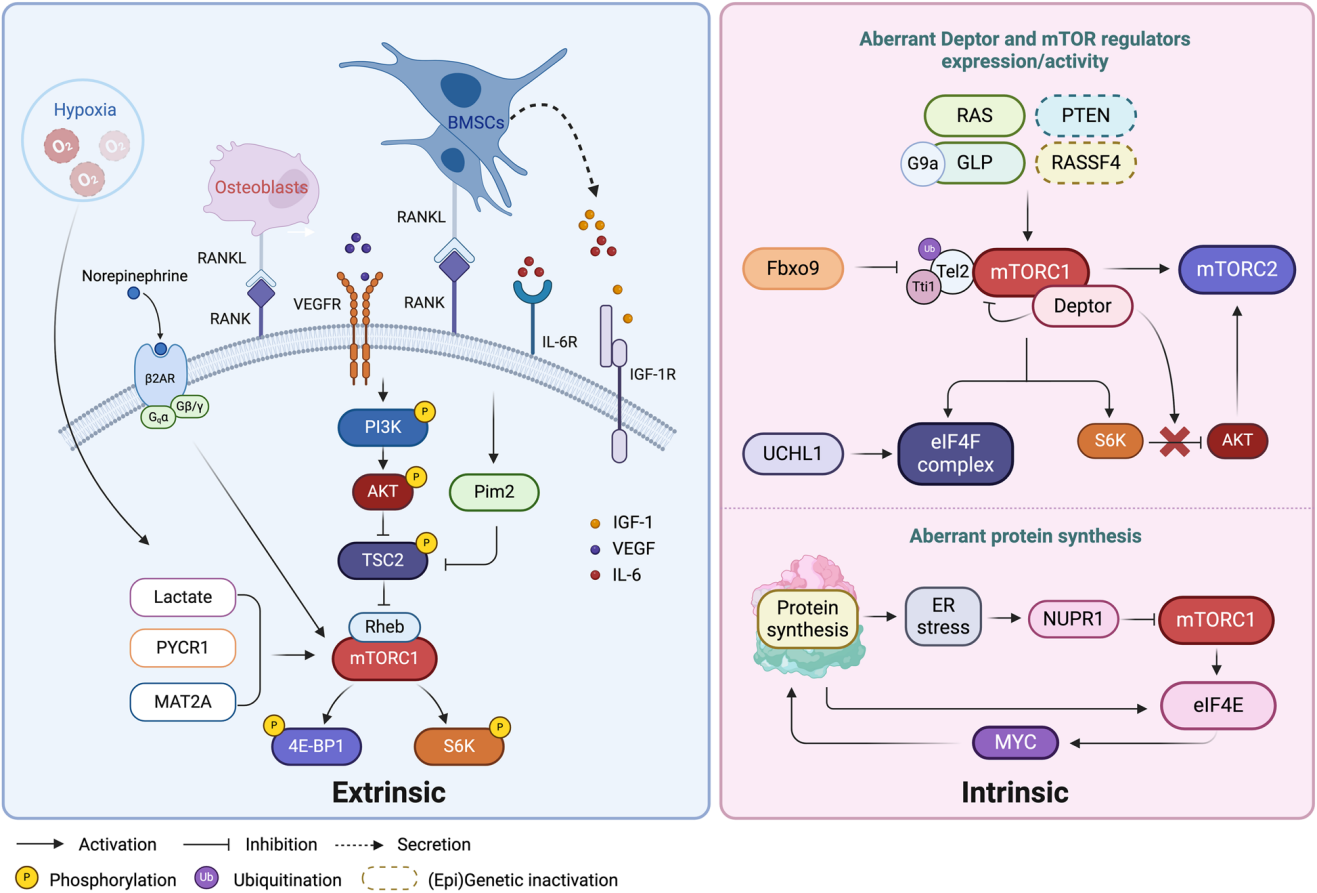
Extrinsic and intrinsic factors regulating mTOR signaling in MM. Extrinsic factors: The myeloma growth factors IL-6, VEGF and IGF-1, which are abundantly present in the BM microenvironment, all induce mTORC1 activation via PI3K/AKT signaling. In addition, cell–cell contact with BMSC and osteoblasts via RANK-RANKL binding also activates PI3K/AKT/mTOR signaling in the MM cells. In addition, Pim2 overexpression, triggered by cytokines or cell–cell contact, also leads to mTORC1 activation via phosphorylating TSC2, while the hypoxic microenvironment mediates mTORC1 activity by regulating lactate, PYCR1 and MAT2A levels. Finally, β2AR is also involved in mTOR activation. Intrinsic factors: Deptor overexpression in MM cells blocks the inhibitory effect of S6K1 on AKT, thereby activating mTORC2. In addition, (Epi)genetic alterations, such as RAS mutationsPTEN depletionoverexpression of G9a/GLP and epigenetic silencing of RASSF4, all support enhanced mTORC1 signaling. Additionally, UCHL directly promotes the assembly of eIF4F. In contrast, Fbxo9 overexpression suppresses mTORC1 signaling by selectively targeting Tel2 and Tti1 in mTORC1 for degradation, which again releases mTORC2 from the negative feedback loop with mTORC1, leading to its activation. To maintain a high rate of protein synthesis, eIF4E is overexpressed in MM. Overexpressed eIF4E in turn promotes protein synthesis by upregulating MYC. Moreover, ER stress, induced by this massive protein synthesis, suppresses mTORC1 signaling via upregulating NUPR1. IL-6, Interleukin 6; IGF-1, Insulin-like growth factor-1; BM, Bone marrow; mTORC1, mTOR complex 1; mTORC2, mTOR complex 2; BMSC, Bone marrow stromal cells; RANK, Receptor activator of nuclear factor-kB; RANKL, RANK Ligand; mTOR, Mammalian target of rapamycin; PYCR1, Pyrroline-5-carboxylate reductase 1; MAT2A, Methionine Adenosyltransferase 2α; PI3K, Phosphatidylinositol-3-kinase; AKT, Protein kinase B; 4E-BP1, Eukaryotic translation initiation factor 4E-binding protein 1; S6K1, Ribosomal S6 kinase; Deptor, DEP-domain containing mTOR-interacting protein; PTEN, Phosphatase and tensin homolog deleted from chromosome 10; GLP, G9a-like protein; RASFF4, Ras-association domain family member 4; Tel2, Telomere maintenance 2; Tti1, Tel2 interacting protein 1; Fbxo9, F-box only protein 9; mTORC2, mTOR complex 2; ER, Endoplasmic reticulum; NUPR1, Nuclear protein 1; β2AR: β2 adrenergic receptor; UCHL1: Ubiquitin C-terminal hydrolase L1; TSC2: Tuberous sclerosis; eIF4F, Eukaryotic translation initiation factor 4F; eIF4E, Eukaryotic translation initiation factor 4E

Over the years, dysregulation of mTOR has been associated with many diseases, such as diabetes, neurological disorders, and cancer (including MM) [88]. mTOR signaling is influenced in MM by numerous factors (Fig. 3), which can be subdivided in extrinsic, BM microenvironment-derived factors and intrinsic, cell-autonomous factors.

#### Extrinsic, BM microenvironment-derived factors

IL-6 and IGF-1, as prominent MM growth factors secreted by the BM microenvironment, activate the mTOR signaling pathway in myeloma cells, as evidenced by phosphorylation of S6K1 and 4E-BP1 upon stimulation [89–91]. IL-6-induced S6K1 activation can be inhibited by rapamycin, the ERK inhibitor PD98059, as well as a dominant negative mutant of AKT, suggesting that both ERK and PI3K/AKT are required for IL6-induced mTOR/S6K1 activation. In contrast, IL-6-induced phosphorylation of 4E-BP1 is only inhibited by rapamycin and the dominant negative AKT, indicating that PI3K/AKT/mTOR is sufficient for 4E-BP1 phosphorylation in MM. Similarly, for IGF-1, phosphorylation of S6K1 and 4E-BP1 can be abolished by the PI3K inhibitor LY294002 and rapamycin [90, 92]. Importantly, IL-6 and IGF-1-induced MM cell growth can also be blocked by activation of AMPK, using metformin or the AMPK activators 5-aminoimidazole-4-carboxamide riboside (AICAr) and D942, leading to inhibition of mTOR, S6K1 and AKT phosphorylation [93–95]. VEGF will also trigger mTOR activation via AKT. Inhibiting VEGF by bevacizumab blocks both mTOR and the translation initiation factor eIF4E, resulting in G1 cell cycle arrest and cell death [96].

In addition to cytokines/growth factors, the mTOR pathway in MM cells is modulated through various other BM-niche related factors as well. For example, cell–cell interactions with the bone marrow stromal cells (BMSCs) and osteoblasts in the BM microenvironment, mediated by RANK-RANKL binding, foster MM cell survival, growth and drug resistance via c-Src mediated mTOR signaling [97, 98]. Moreover, cytokines such as IL-6 and binding to BMSC will also trigger overexpression of the constitutively active serine/threonine kinase Pim2, which is essential for MM survival by phosphorylating TSC2, leading to mTORC1 activation and signaling [99, 100].

Since the BM environment is hypoxic, it favors metabolic rewiring of MM cells, which is characteristic of a more resistant phenotype. This metabolic rewiring will also affect mTOR signaling in MM cells. We found that under hypoxic conditions, anaerobic glycolysis in MM cells leads to an accumulation of lactate in the BM environment, while metabolic enzymes, such as pyrroline-5-carboxylate reductase 1 (PYCR1) and methionine adenosyltransferase 2α (MAT2A), are also upregulated [101–103]. Blocking lactate secretion via inhibition of the monocarboxylate transporter (MCT), in combination with metformin, led to inhibition of mTOR signaling via activation of upstream AMPK. This in turn reduced protein synthesis, leading to caspase activation [101]. Inhibition of PYCR1 also prevented the activation of the mTOR pathway, which in turn impaired phosphorylation of 4E-BP1, eIF4e and S6K1, as well as their upstream protein PRAS40. Further analysis demonstrated that PYCR1 inhibition also reduced cellular uptake of puromycin, confirming that protein synthesis was inhibited [102]. Similarly, MAT2A inhibition also inactivated the mTOR-4E-BP1 pathway, accompanied with a decrease in protein synthesis, again resulting in MM cell death [104].

Finally, the sympathetic nervous system forms a regulatory component of the BM whereby sympathetic nerve fibers form a niche to regulate hematopoiesis during homeostasis and stress. These fibers release norepinephrine that will bind to the β2 adrenergic receptor (β2AR), found on the different cell types in the BM [105]. We found that β2AR is a poor prognostic factor in MM and that the β2AR blocker propranolol inhibited mTOR activation which led to increased apoptosis in MM cells [106].

#### Intrinsic, cell-autonomous factors

##### Aberrant Deptor expression/activity

While many human cancers bearing activated mTORC1 and mTORC2 pathways, have downregulated expression of Deptor, in MM cells, the general consensus is that Deptor acts as an oncogene, by compensating for the negative feedback from S6K1 to PI3K, thereby activating AKT [107]. Moreover, in the MM subgroups harboring a cyclin D1/D3 or c-MAF/MAFB translocation, Deptor is highly expressed, suggesting that the MAFB transcription factor regulates Deptor expression [108]. More recently, it was found that in these MM subgroups, Deptor is phosphorylated by ERK at Ser235, which maintains its stability [109]. It has also been shown that Deptor supports the high protein synthesis in MM cells by regulating the transcription of several genes involved in the maintenance of the ER such as ERLIN2, KEAP1, PSEN2 and DERL3 [110]. Accordingly, several studies have shown that inhibition of Deptor leads to increased drug sensitivity in vitro and has potent anti-tumor effects in vivo [107, 111].

##### Aberrant regulators expression/activity

Genetic mutations in the activators or suppressors of mTOR signaling are common in cancers, including MM. KRAS and NRAS are both mutated in approximately 20% of newly diagnosed MM cases and play an important role in the pathogenesis, progression and prognosis of MM. Overexpression of mutated KRAS or NRAS leads to constitutive activation of the mTOR/S6K1 pathway, which was first discovered in the MM cell line ANBL6 [112]. A more recent study showed that both KRAS or NRAS knockdown decrease phosphorylation of the mTORC1 targets, S6K1 and 4E-BP1, in RAS-dependent MM lines. Of note, due to compensatory feedback signaling, NRAS knockdown also increased phosphorylation of the mTORC2 components and its downstream signaling effectors [113]. In addition, the study revealed a possible mechanism for the constitutive activation of mTOR caused by RAS mutations. The mutant isoforms of RAS were demonstrated to coordinate a signaling complex with the amino acid transporter, solute carrier family 3 member 2 (SLC3A2), and mTOR on endolysosomes directly activating mTORC1 by co-opting the amino acid sensing pathways [113]. Many MM cell lines also contain a mutation for phosphatase and tensin homolog deleted from chromosome 10 (PTEN), suggesting a growth advantage for the loss of PTEN. Indeed, these MM cells have constitutive AKT activity and have upregulated mTOR activity. This makes them particularly sensitive to mTOR inhibition, leading to cell cycle arrest [114].

Epigenetic changes can also contribute to aberrant activation of the mTOR pathway in MM cells. We showed that the mTOR pathway is regulated by the histone methyltransferases G9a and G9a-like protein (GLP) in MM. Overexpression of G9a has been reported in several cancers, including MM, correlating with disease progression, metastasis, and poor prognosis [115]. Mechanistic studies by our group revealed that targeting G9a/GLP impaired the activation of the mTOR/4E-BP1 pathway, leading to autophagy-associated apoptosis in MM [116]. Additionally, the tumor suppressive Ras-association domain family (RASSF) proteins are typically silenced in cancer cells through promotor hypermethylation [117]. We demonstrated that RASSF4 is epigenetically silenced in MM cells and that forced expression of RASSF4 increased the anti-MM effect of the MEK inhibitor trametinib via inhibition of the PI3K/mTOR pathway [118].

The germinal center B-cell oncogene ubiquitin C-terminal hydrolase L1 (UCHL1) is a highly expressed oncogene in MM cells, which encodes a deubiquitinating enzyme that regulates the balance between mTOR complexes, by reducing the non-degradative ubiquitination of Raptor in mTORC1, leading to decreased 4E-BP1 phosphorylation, while at the same time promoting mTORC2 assembly. However, in MM, it was found that UCHL1 bypasses the inhibitory effect on 4E-BP1 by directly associating with and promoting the assembly of eIF4F. Depletion of UCHL1 led to cell death both in vitro and in an orthotopic model of myeloma [119].

PI3K/TORC2/AKT signaling and survival of MM cells is also dependent on F-box only protein 9 (Fbxo9) expression, which is highly expressed in primary human MM. F-box proteins form the substrate recognition component of the SCF type of the ubiquitin ligase complex E3, thereby regulating proteolysis through the ubiquitin proteasome system (UPS). In MM, Fbxo9 regulates mTOR signaling through Tel2 and Tti1. In response to serum starvation, overexpression of Fbxo9 attenuates mTORC1 signaling via degradation of Tel2 and Tti1 within mTORC1, whereas mTORC2 signaling is maintained through the relief of the feedback inhibition, leading to constitutive active PI3K/TORC2/AKT signaling and cell survival. By contrast, loss of Fbxo9 increases the cell size and level of cap-dependent translation of a luciferase mRNA via activation of mTORC1 signaling, while BrdU uptake and cell survival were found to be reduced [120].

##### Aberrant protein synthesis

In contrast to other cancer cells, one of the main characteristics of MM cells is the synthesis of large amounts of immunoglobulin (Ig). To cope with this high demand of protein synthesis, eIF4E is overexpressed in myeloma cell lines and primary myeloma cells compared to plasma cells [121]. In a human xenograft mouse model of MM, stable overexpression of eIF4E dramatically accelerated tumorigenesis, whereas eIF4E knockdown impaired tumor progression [121]. Mechanistically, overexpression of eIF4E was shown to control protein synthesis in MM cells by regulating translation of mRNAs with highly complex 5'-untranslated regions, such as c-MYC [122], while eIF4E inhibition reduced the levels of c-MYC and attenuated cell survival and dexamethasone (DEX) resistance [123, 124]. Importantly, hyperactivation of MYC, which is an essential event mediating transformation from the premalignant condition monoclonal gammopathy of undetermined significance (MGUS) to MM, has been proven to be a key factor in the regulation of ribosome biogenesis and protein synthesis [124–126]. MYC directly increases protein synthesis rates by controlling the expression of multiple components of the protein synthesis machinery, including ribosomal proteins (RPs and small or large ribosomal subunits, and their cofactors) and initiation factors of translation, Pol I, Pol III and rDNA [127–129]. Moreover, MYC can stimulate ribosomal RNA (rRNA) modifications by controlling the expression of ribonucleases, rRNA-modifying enzymes, and nucleolar proteins involved in ribosome biogenesis such as NPM, Nop52, Nop56, and DKC1. In addition, MYC protein was shown to translocate to the nucleolus where it can directly regulate rRNA synthesis by binding to E-box elements located in the rDNA promoter [128, 130]. In this way, overexpression of MYC will lead to a substantial increase in nucleolar activity, which is needed to support enhanced protein synthesis [131].

Massive protein synthesis will also lead to high baseline levels of ER stress, triggering protective responses, such as autophagy, in MM cells [132]. Autophagy is usually considered a pro-survival mechanism that cooperates with the UPS to maintain myeloma cell homeostasis, by degrading excessive and misfolded proteins for energy recycling [133]. In MM, ER stress has been shown to promote autophagy by suppressing the PI3K/AKT/mTOR signaling pathway [134]. Nuclear protein 1 (NUPR1) is a stress-related small molecule that is abnormally expressed in MM cells. Previous studies discovered that knockdown of NUPR1 suppresses survival and growth of MM cell lines, by inducing caspase-dependent apoptosis and G0/G1 cell cycle arrest [135]. Later studies suggested that silencing of NUPR1 suppresses autophagy activities and induces autophagy-mediated apoptosis via PI3K/AKT/mTOR signaling in MM cells [136].

### The mTOR pathway as a promising therapeutic target for MM

#### Pre-clinical studies

Since the discovery of the important role of the mTOR pathway in the progression of MM, studies have tested the potential use of mTOR inhibitors for the treatment of MM (Table 1).

**Table 1:** Summary of the pre-clinical studies in MM

Drug	Target	Response	Reference
Rapamycin and its derivatives	mTORC1	Rapamycin shows anti-MM effect with cell cycle arrest, but they can induce drug resistance by activating mTORC2	[44, 114, 137, 138]
Rapamycin + Perifosine	Rapamycin: mTORC1 Perifosine: AKT	Synergistic cytotoxicity	[139]
Rapamycin + Resveratrol	mTORC1/2	Resveratrol enhances the sensitivity of mTORC2 toward rapamycin. Combination inhibits MM1.S cell viability	[140]
Everolimus	mTORC1	Single drug blocks cell cycle, resulting in inhibition of cell proliferation	[141]
Everolimus + Trametinib	Everolimus: mTORC1 Trametinib: MEK	Combination shows synergistic toxicity in all RAS-dependent MM cell lines	[113]
Everolimus + BZ	Everolimus: mTORC1 BZ: Proteasome inhibitor	Synergistic cytotoxicity	[142]
Everolimus + Panobinostat	Everolimus: mTORC1 Panobinostat: HDACi	Synergistic cytotoxicity caused by DNA damage and proliferation suppression	[143]
Everolimus + entinostat	Everolimus: mTORC1 Entinostat: HDACi	Inhibits oncogenic MYC and activates the Cdkn2a tumor suppressor	[144]
Rapamycin + 17-AGG	Rapamycin: mTORC1 17-AAG: HSP90	Synergistically inhibits cell proliferation and induces cell death. Combination also targets BM microenvironment, inhibiting angiogenesis and osteoclast formation	[145]
Rapamycin + Ponatinib	Rapamycin: mTORC1 Ponatinib: Tyrosine kinase	Drug combination blocks OXPHOS and reduces activity of glycolytic enzymes, resulting in synergistic reduction of tumor xenografts without overt toxicity	[146]
TAK-228	mTORC1/2	TAK-228 suppresses survival of MM cell lines and overcomes the BMSC effects	[147]
pp242	mTORC1/2	pp242 leads to stronger cytotoxicity on MM cells and reduces the angiogenic capacity of endothelial cells. pp242 induces synergistic apoptosis when combined with lenalidomide or BZ	[148, 149]
AZD8055	mTORC1/2	AZD8055 induces MM cells apoptosis. Combination of AZD8055 and IGF1R blockers inhibits the phosphorylation of IGF1R and AKT, leading to apoptosis in AKT-expressing MM cell lines	[150]
DCZ0358	mTORC1/2	DCZ0358 has anti-MM activity and antagonizes the BMSC effects. DCZ0358 abrogates the BZ-triggered activation of AKT, leading to the synergistic cytotoxicity in MM cells	[151]
NVP-BEZ235	PI3K/mTOR	NVP-BEZ235 shows high anti-MM activity and induces autophagy. NVP-BEZ235 induces synergistic cell death when combine with BZ, dexamethasone and doxorubicin	[152]

The mTORC1 inhibitor rapamycin (sirolimus) and the rapamycin analogue (rapalog) CCI-779 were the first to be examined in MM, and were shown to have anti-tumor effects in cells containing PTEN mutations by inducing a G1 cell cycle arrest accompanied by reduced c-MYC levels [114]. Moreover, rapamycin and CCI-779 also significantly curtailed the growth of cells containing oncogenic RAS mutants [112]. Using a myeloma xenograft model, CCI-779 was also proven to induce significant, dose-dependent anti-myeloma effects in vivo, along with upregulated p27 and downregulated cyclin D1 and c-MYC levels [137]. However, several studies also revealed major drawbacks of applying rapamycin and CCI-779. Specifically, inhibition of mTORC1 by rapamycin and CCI-779 leads to increased mTORC2 activity, thereby enhancing basal PI3K/AKT signaling resulting in drug resistance [138]. Furthermore, in all RAS-dependent MM cells, inhibition of mTORC1 activity also leads to an enhanced dependence of the MM cells on MEK and ERK signaling, consequently diminishing the drug's effectiveness [113, 153]. This led to the recent discovery of new combination strategies using rapamycin or its analogue for the treatment of MM. For example, combination of rapamycin with perifosine, an AKT inhibitor was found to synergistically induce MM cytotoxicity by overruling the feedback activation of AKT [139]. The insensitivity of mTORC2 to rapamycin could also be bypassed by efficiently blocking both mTORC1 and mTORC2 signaling pathways using a combination of rapamycin with resveratrol, leading to reduced cell viability in the MM1.S cell line [140]. Resveratrol is a polyphenolic compound that has been reported to inhibit proliferation, induce apoptosis, and overcome chemoresistance as a single agent, by interfering with nuclear factor κB (NF-κB) and STAT3 pathways in human MM cells [154]. Also, synergy between everolimus, another rapamycin analogue, and inhibitors targeting classical mitogen-activated protein kinase (MAPK) signaling via MEK and ERK, such as trametinib, was discovered [113, 141]. In addition, rapamycin has been shown to have synergistic antitumor effects when combined with drugs which have already entered the clinic. For one, rapamycinsynergizes with the standard of care (SoC) drug BZ [142, 155]. Another possible combination is with the pan-histone deacetylase inhibitor (HDACi) panobinostat, which lacks therapeutic effectiveness as a single agent despite having promising anti-myeloma capabilities. One of the resistance mechanisms against panobinostat is triggered by overexpression of the C-X-C motif chemokine receptor 4 (CXCR4), which also activates mTOR signaling. Therefore, combining panobinostat with everolimus led to sustained DNA damage and irreversible proliferation suppression, resulting in the abrogation of resistance to HDACi and synergistic cell death [143]. The combination of everolimus and another HDACi entinostat has also been shown to repress oncogenic MYC and activate the Cdkn2a tumor suppressor in MM mouse models [144]. In addition, combination of rapamycin and the heat shock protein 90 (HSP90) inhibitor 17-AAG synergistically inhibited proliferation and survival of MM cells, as well as angiogenesis and osteoclast formation [145]. As mentioned above, one of the mechanisms by which cancer cells can flexibly reprogram their pathways away from specific metabolic blockages is activation of mTOR. Combination of the tyrosine kinase inhibitor ponatinib and rapamycin therefore impaired the production of ATP required for cell proliferation by targeting glycolytic reprogramming and residual OXPHOS [146].

To inhibit mTOR more effectively, a number of ATP-competitive mTOR inhibitors have been developed. Unlike rapamycin and the rapalogs, ATP-competitive mTOR inhibitors target both mTORC1 and mTORC2. TAK-228, also called MLN0128/INK128, is an oral and selective ATP site kinase inhibitor of mTOR. In MM cell lines and primary cells from patients, TAK-228 inhibits the activity of both TORC1 and TORC2, thereby reducing their survival more potently than rapamycin [147]. Pp242 (Tokinib), is another selective ATP-competitive inhibitor of mTOR that has promising anti-cancer activity in several cancer types. Compared to rapamycin, pp242 not only inhibits phosphorylation of mTORC1 substrates S6K1 and 4E-BP1, but also inhibits phosphorylation of AKT. Moreover, pp242 was shown to be more effective than rapamycin for blocking the release of eIF4E from 4E-BP1 [156]. In line with this efficient mTOR inhibition, pp242 strongly impaired survival of primary MM cells isolated from newly diagnosed patients as well as MM cell lines, as evidenced by the induction of caspase-mediated apoptosis. Importantly, the anti-MM effect of pp242 was also validated in vivo [148]. Moreover, since mTORC2 plays a major role in the angiogenic switch in MM, pp242 also reduced the angiogenic capacity of endothelial cells isolated from MGUS and MM patients and enhanced the anti-angiogenic effect of lenalidomide and BZ [148, 149]. Unfortunately, while pp242 can overcome the feedback activation of AKT caused by the inhibition of mTORC1, it still induces activation of ERK, thus limiting its clinical translation [153]. AZD8055 is another ATP-competitive mTOR inhibitor that induces apoptosis in MM cell lines and patient cells. However, in AKT-expressing MM cell lines, AZD8055 also upregulated phosphorylation of insulin-like growth factor 1 receptor (IGF1R), which prevented apoptosis. Combination of AZD8055 and IGF1R blockers was able to inhibit the IGF1-induced phosphorylation of AKT, resulting in apoptosis of the MM cells [150].

Additionally, another novel alkaloid compound, DCZ0358, was synthetized to efficiently inhibit mTOR signaling via dual mTORC1/2 inhibition. This compound has anti-MM potential in both primary and MM cell lines as a single agent. Notably, DCZ0358 also prevented BZ-induced phosphorylation of AKT, resulting in synergistic anti-MM activity [151, 157]. Finally, the dual class I PI3K/mTOR inhibitor NVP-BEZ235 also showed high antitumor activity in MM by regulating the mTOR2-AKT-FOXO3a-BNIP3 pathway [158]. In addition, NVP-BEZ235 induced synergistic cell death in MM cell lines when combined with BZ, dexamethasone and doxorubicin [152].

#### Clinical trials

Given that preclinical studies in MM were able to demonstrate anti-cancer activity of mTOR inhibitors alone or in combination with SoC drugs, several clinical trials evaluated the efficacy of mTOR inhibitors for treating MM (Table 2).

**Table 2:** Summary of the clinical trials in MM

Drug	Target	Phase of trial	Outcomes	Toxicities	Reference
CCI-779	mTORC1	Phase II	16 patients: PR: 1 patient; MR: 5 patients; SD: 6 patients; TTP: 138 days	Fatigue, neutropenia, thrombocytopenia, anemia and stomatitis	[159]
Everolimus	mTORC1	Phase I	17 patients: PR: 1 patient; MR: 1 patient; SD: 8 patients; TTP: 90 days	Pneumonia	[160]
TAK-228	mTORC1/2	Phase I	31 patients: MR: 1 patient; SD: 14 patients	Thrombocytopenia, fatigue, and neutropenia	[161]
CC-223	mTORC1/2	Phase I	1 patient	Hyperglycemia, rash, fatigue, and mucositis	[162]
CCI-779 + BZ	mTORC1 Proteasome inhibitor	Phase I/II	20 patients (Phase I): VGPR: 1 patient; PR: 2 patients; MR: 2 patients; SD: 12 patients 43 patients (Phase II): CR: 2 patients; VGPR: 4 patients; PR: 12 patients; MR: 6 patients; SD: 19 patients	Thrombocytopenia, lymphopenia, neutropenia, leukopenia, and anemia	[163]
Everolimus + lenalidomide	mTORC1 Immunomodulatory drug	Phase I	26 patients: CR: 1 patient; PR: 4 patients; MR: 10 patients; SD: 2 patients	Thrombocytopenia, neutropenia	[164]
Everolimus + bendamustine	mTORC1 Alkylating agent	Phase I	5 patients: VGPR: 1 patient; PR: 3 patients	Lymphopenia, thrombocytopenia, leukopenia, neutropenia and fatigue	[165]

*CR* Complete response, *VGPR* Very good partial response, *PR* Partial response, *SD* Stable disease, *MR* Minimal response, *TTP* Time to progression

CCI-779 was the first mTOR inhibitor to be clinically evaluated in patients with relapsed/refractory (RR) MM. In a phase II trial, 16 patients were enrolled and received monotherapy with CCI-779 (25 mg I.V. weekly). After at least two cycles of treatment, one patient achieved a partial response (PR) and five patients achieved minimal response (MR). Time to progression (TTP) was found to be 138 days. Meanwhile, in patients with a MR or PR, inhibition of p-p70S6K and p-4E-BP1 was observed in the peripheral blood monocytes. Common adverse effects found in clinical trials with mTOR inhibitors were also observed in patients receiving CCI-779 therapy, such as fatigue, neutropenia and thrombocytopenia [159].

Everolimus has been approved by the FDA for the treatment of pancreatic neuroendocrine tumors, advanced renal cell carcinoma, and advanced breast cancer [160]. In MM, 17 patients participated in a phase I clinical trial evaluating oral everolimus therapy in RRMM patients, who had received two or more lines of prior treatment. In all patients, no dose-limiting toxicity was observed, leading to a final dose of 10 mg daily. There were eight patients with stable disease, one patient with minor remission, and one patient in partial remission. However, the median time to disease progression was shorter (only 90 days) compared to patients treated with CCI-779. Notably, only one drug-related adverse event was observed, which was pneumonia [166].

Ghobrial et al. conducted the first clinical trial of the oral TORC1/2 inhibitor TAK-228 in MM patients, as well as patients with non-Hodgkin lymphoma (NHL) or Waldenström's macroglobulinemia (WM). The study evaluated drug safety, tolerability, maximum tolerated dose (MTD), dose-limiting toxicity (DLT), pharmacokinetics, and preliminary clinical activity of TAK-228. Ninety-two percent of the patients reported at least one drug-related toxicity, and the most common grade ≥ 3 drug-related adverse events were thrombocytopenia, fatigue and neutropenia. Of the 31 patients with evaluable responses, only one MM patient had a minimal response, while 14 MM patients had stable disease [161].

CC‐223 is an ATP–competitive inhibitor of mTOR that targets both mTORC1 and mTORC2. CC-223 was shown to be effective in breast cancer, glioma, hepatocellular carcinoma (HCC), non-small cell lung cancer and non-Hodgkin's lymphoma cell lines [167–169]. Twenty-seven patients with advanced solid tumors and one MM patient were enrolled in a phase I clinical trial with CC-223. Only one partial response was observed in breast cancer, while all other patients experienced either stable disease or disease progression. The most common drug-related adverse events were hyperglycemia, fatigue, and diarrhea. Importantly, an association was observed between a CC‐223 response and the reduction in phosphorylation of AKT, 4E-BP1, and S6 ribosomal protein (S6RP) in stimulated B cells, T cells, and monocytes [162].

Overall, the above-described clinical studies with mTOR inhibitors as monotherapy showed only low single agent activity in MM, suggesting the necessity of using alternative doses and combination therapies. Twenty patients with RR MM were enrolled in a phase 1 study to evaluate the combination of CCI-779 and BZ, while forty-three patients were enrolled in the phase 2 of this clinical trial. The percentage of patients with a partial response (or better) in the phase 2 study was 33%. In both studies, the most common treatment-related grade 3–4 adverse events were thrombocytopenia, lymphopenia, neutropenia, leukopenia, and anemia [163]. The combination of everolimus and lenalidomide also showed promising outcomes in a phase I clinical trial in patients with RR myeloma. This drug combination was considered to be relatively safe, with the most common observed grade 3 or 4 adverse events being thrombocytopenia and neutropenia. Of the twenty-six patients included in the evaluation, twenty-three were considered as evaluable responses, with one patient showing a complete response (CR), four patients showing PR, and ten patients achieving MR, accounting for an overall response rate of 65%. Analysis of the plasma samples obtained before and after treatment showed that p-p70S6K was downregulated, and more importantly, responders expressed higher basal levels of mTOR pathway-related proteins compared to non-responders [164]. Recently, another phase I study of everolimus and bendamustine in patients with RR MM also showed promise, resulting in an 80% overall response rate with only mild adverse events. Eighteen adult patients with RR lymphoid malignancies were eligible. Of the five patients with MM, three patients showed a PR, while one patient achieved a very good partial response (VGPR) [165].

## Conclusions and future perspectives

mTOR has been identified as a central regulator of multiple signaling pathways that work together to integrate growth factor, nutrient, and amino acid signals, thereby modulating the expression and activity of proteins involved in protein synthesis, cell growth and cell survival. While mTOR is a key signaling pathway in MM, most MM studies limit their study to simply demonstrate that different types of inhibitors lead to a reduction in mTOR without further evaluation of the up- or downstream components. Here we aimed to highlight those studies with demonstrated impact on downstream signaling, especially since recent studies using advanced techniques have identified the different components of mTORC1 and mTORC2, contributing to a new perspective on the mechanism of mTOR hyperactivation and the resultant consequences in tumor cells. The recent identification of the novel regulators, such as Tel2 and Tti1, and their function further strengthens the idea that mTOR complexes are intricate assemblies. Future research should further delve into the detailed effects of upstream factors on specific components of the mTOR complexes, aiming to achieve a more profound understanding of its assembly and activation.

While inhibitors targeting the mTOR pathway have achieved significant therapeutic effects in solid tumors (including renal and breast cancer), results of clinical trials testing mTOR inhibitor monotherapies for the treatment of MM have been mostly disappointing. There are several (possible) explanations for these disappointing results. First, the mTOR pathway is a complicated pathway that provides several potential targets, and it remains unclear if one or more targets need(s) to be inhibited in MM and whether these should be simultaneously or rather sequentially. Second, feedback loops contribute to the resistance to mTOR inhibitors. Third, the heterogeneity often observed in MM is likely to make the mTOR activation patterns even more diverse. Finally, high doses inducing adverse effects following treatment with mTOR inhibitors may be due to the critical roles of mTOR in immunity, which is still less understood in MM. Therefore, it would be interesting to investigate mTOR signaling networks in different myeloma tumor clones, as well as in their neighboring cells, including immune cells and BM stromal cells. This will provide crucial mechanistic information to guide the rational development of novel combinations of mTOR inhibitors with chemotherapeutic agents and/or targeted drugs to improve survival of MM patients. Notably, multiple combinations of targeted therapy strategies are suitable only for specific cancer types, as seen with NVP-BEZ235 plus abiraterone acetate (a CYP17 inhibitor), which is primarily used in treating castration-resistant prostate cancer [170, 171]. Hence, it will be crucial to identify predictive biomarkers in MM to guide the stratification of patients in clinical trials and identify those likely to benefit the most from treatment with mTOR inhibitors.

## Data Availability

No datasets were generated or analysed during the current study.

## Abbreviations

MM: Multiple Myeloma
mTOR: Mammalian target of rapamycin
BM: Bone Marrow
MGUS: Monoclonal gammopathy of undetermined significance
PI: Proteasome inhibitors
IMiD: Immunomodulatory drugs
ASCT: Autologous stem cell transplantation
OS: Overall survival
CAR-T: Chimeric antigen receptor T
mTORC1: MTOR complex 1
mTORC2: MTOR complex 2
mLST8: Mammalian Lethal with Sec-13 protein 8
Deptor: DEP-domain containing mTOR-interacting protein
PRAS40: Proline-rich AKT substrate 40 kDa
Raptor: Regulatory-associated protein of mTOR
mSIN1: Mammalian stress-activated map kinase-interacting protein 1
Rictor: Rapamycin-insensitive companion of mTOR
Protor: Protein observed with rictor
NTD: N-terminal domain
RBD: RAS-binding domain
CRIM: Conserved region in the middle
PH: Pleckstrin homology
Tel2: Telomere maintenance 2
Tti1: Tel2 interacting protein 1
PI3K: Phosphatidylinositol-3-kinase
PIP3: Phosphatidylinositol-3, 4, 5-triphosphate
PDK1: Phosphoinositide-dependent kinase 1
AKT: Protein kinase B
TSC: Tuberous sclerosis
GAP: GTPase-activating protein
USP4: Ubiquitin specific peptidase 4
Arf1: ADP-ribosylation factor 1
AMPK: Adenosine 5'-monophosphate-activated protein kinase
HIF-1: Hypoxia inducible factor 1
BNIP3: BCL2-interacting protein 3
DDIT4/REDD1: DNA damage inducible transcript 4
IRS1: Insulin receptor substrate 1
GPCR: G-protein coupled receptors
PKA: CAMP-dependent protein kinase
ER: Endoplasmic reticulum
4E-BP1: Eukaryotic translation initiation factor 4E-binding protein 1
S6K1: Ribosomal S6 kinase 1
eIF4E: Eukaryotic translation initiation factor 4E
PDCD4: Programmed cell death protein 4
SKAR: S6k1 Aly/REF-like substrate
PABP: Poly(A)-binding protein
rpS6: Ribosomal protein S6
Paip1: PABP-interacting protein 1
eEF2k: Eukaryotic elongation factor 2 kinase
Pol I/III: RNA polymerase I/III
5’-TOP: 5’-Terminal oligopyrimidine
LARP1: La-related protein 1
EMT: Epithelial-mesenchymal transition
IL-6: Interleukin 6
IGF-1: Insulin-like growth factor-1
ERK: Extracellular signal-regulated kinase 1
AICAr: 5-Aminoimidazole-4-carboxamide riboside
VEGF: Vascular endothelial growth factor
BMSCs: Bone marrow stromal cells
RANK: Receptor activator of nuclear factor-κB
RANKL: RANK Ligand
MCTs: Monocarboxylate transporters
PYCR1: Pyrroline-5-carboxylate reductase 1
BZ: Bortezomib
MAT2A: Methionine adenosyltransferase 2α
PTEN: Phosphatase and tensin homolog deleted from chromosome 10
GLP: G9a-like protein
RASSF: Ras-association domain family
Fbxo9: F-box only protein 9
UPS: Ubiquitin proteasome system
Ig: Immunoglobulin
DEX: Dexamethasone
rRNA: Ribosomal RNA
NUPR1: Nuclear protein 1
NF-κB: Nuclear factor κB
MAPK: Mitogen-activated protein kinase
SoC: Standard of care
HDACi: Histone deacetylase inhibitor
CXCR4: C-X-C motif chemokine receptor 4
HSP90: Heat shock protein 90
IGF1R: Insulin-like growth factor 1 receptor
RR: Relapsed/refractory
PR: Partial response
MR: Minimal response
TTP: Time to progression
NHL: Non-Hodgkin lymphoma
WM: Waldenström’s macroglobulinemia
MTD: Maximum tolerated dose
DLT: Dose-limiting toxicity
HCC: Hepatocellular carcinoma
S6RP: S6 ribosomal protein
CR: Complete response
VGPR: Very good partial response
β2AR: β2 Adrenergic receptor
UCHL1: Ubiquitin C-terminal hydrolase L1
MEK: Mitogen-activated protein kinase/ERK kinase
